# Novel Semi-Interpenetrated Polymer Networks of Poly(3-Hydroxybutyrate-co-3-Hydroxyvalerate)/Poly (Vinyl Alcohol) with Incorporated Conductive Polypyrrole Nanoparticles

**DOI:** 10.3390/polym13010057

**Published:** 2020-12-25

**Authors:** José Luis Aparicio-Collado, Juan José Novoa, José Molina-Mateo, Constantino Torregrosa-Cabanilles, Ángel Serrano-Aroca, Roser Sabater i Serra

**Affiliations:** 1 Centre for Biomaterials and Tissue Engineering, Universitat Politècnica de València, 46022 València, Spain; joapcol@upvnet.upv.es (J.L.A.-C.); jmmateo@fis.upv.es (J.M.-M.); ctorregr@fis.upv.es (C.T.-C.); 2 University of Applied Sciences Technikum Wien, 1200 Vienna, Austria; jj.novoa10@gmail.com; 3 Biomaterials and Bioengineering Lab, Centro de Investigación Traslacional San Alberto Magno, Universidad Católica de Valencia San Vicente Mártir, 46001 Valencia, Spain; 4 CIBER-BBN, Biomedical Research Networking Centre in Bioengineering, Biomaterials and Nanomedicine, 46022 València, Spain

**Keywords:** nanocomposite, semi-interpenetrating network, hydrogel, electroactive biomaterial, conductive polymer, poly (3-hydroxybutyrate-co-3-hydroxyvalerate), poly (vinyl alcohol), polypyrrole nanoparticles, tissue engineering

## Abstract

This paper reports the preparation and characterization of semi-interpenetrating polymer networks (semi-IPN) of poly(3-hydroxybutirate-co-3-hydroxyvalerate), PHBV, and poly (vinyl alcohol), PVA, with conductive polypirrole (PPy) nanoparticles. Stable hybrid semi-IPN (PHBV/PVA 30/70 ratio) hydrogels were produced by solvent casting, dissolving each polymer in chloroform and 1-methyl-2-pyrrolidone respectively, and subsequent glutaraldehyde crosslinking of the PVA chains. The microstructure and physical properties of this novel polymeric system were analysed, including thermal behaviour and degradation, water sorption, wettability and electrical conductivity. The conductivity of these advanced networks rose significantly at higher PPy nanoparticles content. Fourier transform infrared spectroscopy (FTIR) and calorimetry characterization indicated good miscibility and compatibility between all the constituents, with no phase separation and strong interactions between phases. A single glass transition was observed between those of pure PHBV and PVA, although PVA was dominant in its contribution to the glass transition process. Incorporating PPy nanoparticles significantly reduced the hydrogel swelling, even at low concentrations, indicating molecular interactions between the PPy nanoparticles and the hydrogel matrix. The PHBV/PVA semi-IPN showed higher thermal stability than the neat polymers and PHBV/PVA blend, which also remained in the tertiary systems.

## 1. Introduction

Polyhydroxyalkanoates (PHA) are a family of biodegradable aliphatic polyesters synthesised by bacteria and archaea as an intracellular carbon and energy storage compound [1]. The PHA copolymer poly (3-hydroxybutyrate-*co*-3-hydroxyvalerate) (PHBV) has emerged as a promising material for biomedical applications, due to its null cytotoxicity and its current large-scale production [2,3,4]. PHBV’s biocompatibility and biodegradability render it an excellent biomaterial with a wide range of applications in the fields of cardiovascular stents [5], controlled drug release [6,7], absorbable surgical sutures [8], tissue patches, biodegradable implants, and tissue engineering scaffolds [9]. It has also been proposed as a ‘green’ dielectric material (lead- and halogen-free) for capacitors in a wide range of applications, such as electric power circuits for implantable medical devices [10]. As a hydrophobic polymer, PHBV possess outstanding mechanical properties with a Young’s modulus of about 1–5 GPa [11]. However, despite the excellent properties of PHBV, its biomedical use is still limited due to its lack of water sorption, brittleness and low thermal stability [12,13].

On the other hand, hydrophilic polymers networks, commonly known as hydrogels, are particularly useful and advantageous materials due to their capacity of being able to absorb and retain large amounts of water within their polymer network without being dissolved in aqueous solutions [14,15]. These water sorption properties have made these three dimensional networks very promising biomaterials for a wide range of applications, such as drug delivery, tissue engineering [16] and wound dressing [17]. More specifically, injectable hydrogels have been used in bone tissue engineering as carriers for bioactive factors, such as growth factors, that will be released after the hydrogel degradation [18]. The hydrophilic polymer poly (vinyl alcohol) (PVA) has been extensively studied in biomedicine applications due to its excellent water sorption properties, biocompatibility and biodegradability [19,20]. PVA can be crosslinked by multiple methods, ranging from chemical agents like glutaraldehyde (GA) and other monoaldehydes to electron beams, γ-irradiation or physical crosslinking due to crystallite formation [21]. PVA hydrogels have been widely used in tissue engineering [22]: neurogenesis and osteogenesis promotion [19], cardio myoblasts culture [23] and fibroblasts culture for knee implants [24]. However, like most hydrogels, the mechanical properties of PVA are poor.

Multipolymer networks, also known as hybrid networks, such as semi-interpenetrating polymer networks (semi-IPNs), interpenetrating polymer networks (IPNs) and the so-called ‘double networks’ (DN), with a particular IPN structure, have emerged as an effective alternative to overcome the limitations of pure polymers [25,26]. In this regard, semi-IPNs and IPNs can lead to reinforced interwoven polymer networks. An IPN can be defined as a polymeric matrix comprising two crosslinked networks interlaced on a molecular scale, whilst a semi-IPN is a matrix comprising only one crosslinked network, in which the linear or blanched polymer penetrates the network on a molecular scale, i.e., the linear or branched macromolecules are dispersed into the polymer network [27]. Mechanically enhanced DNs, characterized by good mechanical properties, are formed by a first densely cross-linked ionic hydrogel and a neutral loosely cross-linked network [26]. These DN hydrogels are composed of crosslinked polymers without covalent bonds between the polymer networks. Nevertheless, at least one of these polymer networks is synthesized and/or crosslinked within the presence of the second network [28,29].

These hybrid structures combine the advantageous properties of each polymeric component leading to a new material with properties that may be different from those of the pure components [27,30]. Depending on the nature of the constituent polymers, the combination of synthetic and natural polymers, with hydrophobic and hydrophilic properties, can result in a broader range of properties and applications [25,31].

Hydrogel properties can be effectively modified by preparing hybrid networks (IPNs or semi-IPNs) due to their having better properties in a blend that those of their individual components [25,26]. In aqueous environments, hydrogels formed by a crosslinked hydrophilic polymer with chains of a hydrophobic polymer dispersed into the network (i.e., a semi-IPN hydrogel) can be an interesting approach to combine the properties of both components. The hydrogel provides hydration while preventing dissolution of the hydrophilic polymer and the hydrophobic polymer provides improved physical properties. Semi-IPNs hydrogels synthesised from biocompatible polyurethane and acrylamide monomer [28], PVA with poly(caprolactone) [11] or with poly(acrylamide-co-styrene) [32] with enhanced physical and biological properties have been reported for biomedical applications (particularly tissue engineering and drug release). However, no semi-IPNs of PHBV/PVA have been reported in the literature so far.

Conductive polymers (CPs) include a family of polymers with a mixture of the properties of metals (ability to conduct electrical charges) and conventional polymers (ease of synthesis and processing) [33]. Conductive and semiconductive polymers, either alone or in combination with other polymers, have recently attracted growing interest in the field of biomedicine and regenerative medicine, particularly as cell substrates for cell culture with or without external electrical stimulation or conducting polymer-based biosensors [34,35]. Several studies have shown that cell substrates with conductive (or electroactive) properties can enhance bioactivity, promoting cell response in terms of proliferation and differentiation [36,37]. Recently, electroactive composite scaffolds have been prepared to control the release of osteoinductive factors by external electrostimulation [38]. In addition, infection produced by microbes have become a social problem due to the rise of antibiotic-resistant microorganisms [39]. Novel antimicrobial agents, among them conducting polymers, have been applied as a new tool to address this problem [40].

Conductive polymers such as polypyrrole (PPy), polyaniline and the family of polythiophene polymers have been reported for biomedical applications, both as bioactive and biocide agents [41,42]. PPy is a polymer with good electrical conductivity, insoluble in water, with proven in vitro and in vivo biocompatibility [41] and antimicrobial activity [40]. However, problems related to their poor solubility and non-degradability can limit their use. One way to overcome these problems is the preparation of materials from blends and composites based on CPs and degradable polymers: such as PHBV, poly(caprolactone), polylactic acid, gelatin, or collagen [33,43,44,45,46], or also grafts, such as PPy grafted with oligo-3-hydroxybutyrate pendants reported in refs. [47,48].

In this study, we explore the preparation and characterization of reinforced semi-IPN hydrogels based on PHBV/PVA with incorporated conductive nanoparticles of PPy with the aim of obtaining novel polymeric biomaterials with intrinsic bioactive and antimicrobial properties for potential application in the field of tissue engineering. The semi-IPN was prepared with the hydrophobic PHBV and the hydrophilic PVA in a 30/70 ratio in order to provide structural reinforcement while preserving the major characteristics of hydrogels. To increase blend compatibility and simultaneously avoid PVA dissolution when the substrates are introduced into aqueous environments (such as a culture medium), PVA was chemically crosslinked with GA to form an insoluble 3D network, in which the PHVB chains were entwined within the PVA network. Neat PVA, PHBV and a 30/70 PHBV/PVA blend were also prepared as control materials. Since these semi-IPN networks lack conductive properties, the strategy of introducing conductive PPy nanoparticles is proposed here. Several PPy nanoparticles (diameter 300–500 nm) contents, ranging from 0 to 15% *wt/wt*, were incorporated into the substrates. The morphology and physicochemical properties (swelling, wettability and thermal behaviour/degradation) of these novel semi-IPN hydrogels were determined in this study. Semi-IPNs filled with conductive PPy nanoparticles were also analysed in terms of their microstructural properties and electrical behaviour.

## 2. Materials and Methods

### 2.1. Materials

Poly (3-hydroxybutirate-*co*-3-hydroxyvalerate), PHBV, with 2% wt of 3-hydroxyvalerate (Mw 410,000 g/mol) was supplied by Goodfellow (Huntingdon, UK), product code BV336010. Poly (vinyl alcohol), PVA, (Mw 13,000–23,000, 87–89% hydrolyzed, product code 363170), polypyrrole nanoparticles (PPy) doped with an organic sulfonic acid as dopant (conductivity 10–50 S/cm and stable up to 290 °C, product code 577030) and 1-metil-2-pyrrolidone (NMP) were purchased from Sigma Aldrich-Merck (St. Louis, MO, USA). PVA with a low molecular weight was selected because it biodegrades faster [49]. However, PVA with lower molecular weight exhibits lower mechanical performance [50]. Thus, since the mechanical properties and thermal stability of PHBV increases rapidly with molecular weight [51] we selected a PHBV with a high molecular weight to enhance the physical properties of PVA. Chloroform (99–99.6% pure), glutaraldehyde (GA) solution 25 wt.% in H_2_O, methanol and sulfuric acid (95–98% extra pure) and acetic acid glacial (99% extra pure) were supplied by Scharlab (Barcelona, Spain). All reagents were used as received.

### 2.2. Preparation of Neat Polymers and 30/70 PHBV/PVA (Poly (3-Hydroxybutyrate-Co-3-Hydroxyvalerate)/Poly (Vinyl Alcohol)) Blend Films

PHBV, PVA and 30/70 PHBV/PVA blend films were prepared by solvent casting. PHBV was dissolved in chloroform (3% *wt/wt*) at 50 °C with constant stirring for 120 min. PVA was dissolved in NMP (5% *wt/wt*), also with constant stirring for 120 min, gradually raising the heating plate temperature from 25 °C to 150 °C. After complete dissolution, the neat polymer solutions were poured into petri dishes. Polymer films were obtained after solvent evaporation at room temperature for 24h (neat PHBV) and in an air oven at 60 °C for 72 h (PVA). PHBV/PVA blends were prepared by mixing PHBV/chloroform and PVA/NMP solutions (30/70 *wt/wt* ratio) with magnetic stirring for 24 h at 50 °C. The mixed solution was poured into a Petri dish and PHBV/PVA films were obtained after 24 h of chloroform evaporation at room temperature followed by 72 h in an air oven at 80 °C to evaporate the NMP. All the prepared films were dried at 60 °C under vacuum to constant weight to completely remove all traces of solvent. The thickness of the samples (≈300 µ) was measured by a digital calibre (Acha, Eibar, Spain).

### 2.3. Preparation of PHBV/PVA Semi-Interpenetrating Polymer Network (IPN) (PVA Crosslinking)

PVA was crosslinked to achieve aqueous stability, forming a semi-IPN hydrogel with (uncrosslinked) PHBV. The crosslinking of the PVA phase in the semi-IPN was performed according to the procedure reported by Rudra et al. [52], using GA as crosslinker (4% *wt/wt* GA with respect of the total PVA content). To accelerate the rate of the crosslinking reaction, sulfuric acid as catalyst, methanol as a quencher and acetic acid as pH controller were used. Four aqueous solutions were prepared: solution 1 with 25% GA (crosslinker) as a crosslinking solution, solution 2 with 10% sulfuric acid, solution 3 with 10% acetic acid and solution 4 with 50% methanol. These reagent solutions were mixed in a 2:1:3:2 volumetric ratio and then transferred to the mixed PHBV/chloroform and PVA/NMP solution with vigorous stirring. Using the same procedure as that used for 30/70 PHBV/PVA blends, the mixed solution was casted and left for 24 h at room temperature to evaporate chloroform followed by 72 h in an air-oven at 80° to completely evaporate NMP. The crosslinked films were then immersed twice in Mili-Q water at 37 °C (24 h) to remove any GA residue and left 48 h at room temperature to evaporate water. Finally, the semi-IPN films were dried at 60 °C under vacuum to constant weight to remove residual moisture.

### 2.4. Electroconductive Particles Embedding

Four different concentrations of PPy (2, 5, 10 and 15% *wt/wt* based on the mass of the polymeric matrix) were introduced in the 30/70 PHBV/PVA matrix. First, PPy particles were dispersed in NMP solvent by sonication for 30 min, after which PVA was dissolved in the NMP-PPy suspension, and the PHBV/PVA/PPy semi-IPN films were prepared following the protocols described in Section 2.2 and Section 2.3.

The notation and sample compositions are included in Table 1.

### 2.5. Characterisation Techniques

#### 2.5.1. Electron Microscopy

The surface and cross-section morphology of the samples were analysed in a GeminiSEM 500 high-resolution field-emission scanning electron microscope (HR-FESEM) (Carl Zeiss Microscopy, Jena, Germany) with an accelerating voltage of 0.8–1.0 kV. The samples were coated with a platinum layer by an EM MED020 sputter coater (Leica, Wetzlar, Germany). The cross-section was observed in samples previously immersed in liquid N_2_ and cryofractured. The morphology of PPy nanoparticles was observed by HR-FESEM and a TEM (transmission electron microscope JEM 2100F operated at 200 kV, JEOL, Tokyo, Japan). Previously, the nanoparticles were dispersed in an ultrasound bath for 1h. One drop was then placed on a SEM sample holder and carbon-coated TEM grid for 2 h to ensure complete drying. An estimation of the size distribution of PPy nanoparticles was obtained from HR-FESEM micrographs with ImageJ software. The diameter of about 100 PPy nanoparticles (only those that show clear spherical shape) was measured to obtain the average diameter and diameters’ distribution.

#### 2.5.2. Fourier Transform Infrared Spectroscopy (FTIR)

Functional groups in the systems were determined by Fourier transform infrared spectroscopy (FTIR, Bruker Optics FTIR Alpha II). Samples underwent 24 scans at room temperature, and FTIR spectra were collected in transmittance mode from 4000 to 400 cm^−1^ at a resolution of 2 cm^−1^.

#### 2.5.3. Swelling Assay

Swelling experiments were performed gravimetrically in crosslinked samples: PHBV/PVA E and semi-IPNs with 2, 5, 10 and 15% of PPy nanoparticles with PVA E as reference. Square samples (10 × 10 mm and thickness ≈ 300 µm) were vacuum-dried at 60 °C and immersed in Mili-Q water at 37 °C until equilibrium. Redundant surface water was removed by filter paper. Experiments were performed in triplicate to ensure reproducibility.

Samples were weighed before (*W*_0_) and after (*W*_1_) swelling, and the swelling degree (*W_eq_*) was calculated as:

(1) $$W_{eq}\left(\%\right)=\;\;\frac{W_{1}-W_{0}}{W_{0}}100$$

#### 2.5.4. Surface Wettability

The surface wettability was determined by water contact angle (WCA) on the surface of films (thickness ≈ 300 µm) using the sessile drop method. Hydrophobic PHBV, hydrophilic PVA (non-crosslinked and crosslinked) were used as reference. 30/70 PHBV/PVA blend and semi-IPNs with and without PPy nanoparticles were analysed. To measure the contact angles, a 3 μL Mili-Q water drop was deposited onto the surface and stabilization was allowed (~10 s) at room temperature. WCA was measured by using an optical contact angle and contour analysis system (Dataphysics OCA 20, Charlotte, NC, USA). All measurements were performed in triplicate.

#### 2.5.5. Differential Scanning Calorimetry (DSC)

Differential scanning calorimetry (DSC) analysis was carried out on a PerkinElmer DSC 8000 (PekinElmer, Waltham, MA, USA) under a flowing nitrogen atmosphere (20 mL/min). After erasing the effects of any previous thermal history by heating at 220 °C, the samples were subjected to a cooling scan down to −20 °C, followed by a heating scan from this temperature to 220 °C, both at 20 °C/min. The glass transition temperature, *T_g_*, was calculated from the heating scan as the inflexion point of the specific heat capacity, *C_p_*, vs. temperature, which coincides with a maximum in the temperature derivative (*dc_p_/dT*). The width of the glass transition, Δ*T_g_*, was obtained by the intersections of the tangent line at the inflexion point with the extrapolated glass and liquid lines. Finally, the specific heat capacity increment at the measured glass transition, Δ*c_p_(T_g_)*, was determined as the difference in heat capacity between the extrapolated liquid and glass lines at *T_g_*.

#### 2.5.6. Thermogravimetric Analysis (TGA)

Thermogravimetric analysis (TGA) was used to study the thermal decomposition of each sample related to gradual temperature increases. Vacuum-dried samples (5–10 mg weight) were heated from 30 to 600 °C at a rate of 10 °C/min in a Mettler Toledo TGA 2 (SF) system (Mettler Toledo, Columbus, OH, USA). The mass of the samples was constantly measured as a function of temperature.

#### 2.5.7. Conductivity Analysis

The electrical sheet resistance (*R_S_*) of circular film samples of approximately 10 mm diameter was measured on a T2001A3-EU four-point probe system (Ossila Limited, Sheffield, UK). The electrical conductivity (*σ*) in S/m was calculated according to the following formula:

(2) $$\sigma =\;\frac{1}{R_{S}\times l}$$

where *l* is the film thickness, which was measured by a digital caliper (Acha, Spain). The measurements were performed in triplicate in order to ensure reproducibility.

#### 2.5.8. Statistical Analysis

Statistical analyses were carried out on swelling degree, static contact angle and conductivity by one-way analysis of variance (ANOVA) tests on all samples using GraphPad Prism 6.0 software. Data were presented as mean ± standard deviation. If significant differences were noted between the samples, Tukey tests were used for pairwise comparisons with a 95% confidence interval (*p* < 0.05).

## 3. Results and Discussion

To obtain this novel hydrogel with improved structural enhancement, the semi-IPN PHBV/PVA was prepared with 30% of PHBV and 70% of PVA and subsequent crosslinking of PVA with GA. This ratio, with a high percentage of the hydrophilic polymer, was chosen to achieve the mechanical reinforcement provided by the hydrophobic PHBV but maintaining the water absorption capacity characteristic of hydrogels. As will be shown in this section, this ratio allowed to obtain a hybrid network with hydrogel characteristics. In PHBV/PVA blends with PVA percentage lower than 70%, the PVA crosslinking was not completely effective and the samples were partially dissolved after immersion in water.

### 3.1. Microstructure and Fourier Transform Infrared Spectroscopy (FTIR) Analysis

**Figure 1:**
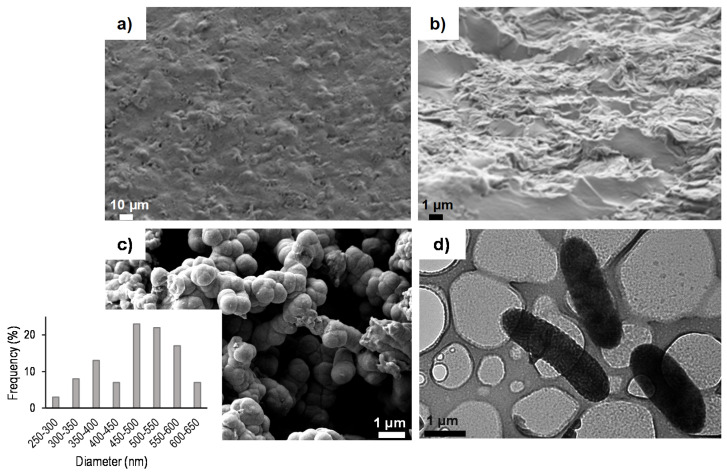
High-resolution field-emission scanning electron microscope (HR-FESEM) photomicrographs of semi-interpenetrating polymer network (IPN) poly (3-hydroxybutyrate-co-3-hydroxyvalerate)/poly (vinyl alcohol) (PHBV/PVA E). (**a**) Surface and (**b**) cross-section. (**c**) HR-FESEM and (**d**) TEM photomicrographs of polypirrole (PPy) nanoparticles. Inset in (**c**) shows the diameters’ distribution.

Figure 1 shows the HR-FESEM images of the surface and cross-section (from cryogenic fracture) of the PHBV/PVA semi-IPN hydrogels. A homogeneous structure can be seen in both the surface and cross-section (Figure 1a,b). The cross-section had some areas with a brittle fracture and few threads were identified between the brittle areas, as reported for semicrystalline polymers [53]. Figure 1c,d present HR-FESEM and TEM images, respectively, of the PPy nanoparticles as received. The PPy nanoparticles show a rod-like morphology (see the TEM image), with an average diameter of 480 nm (in agreement with previous results [54]). The diameter distribution of the nanoparticles is shown in the inset of Figure 1c.

**Figure 2:**
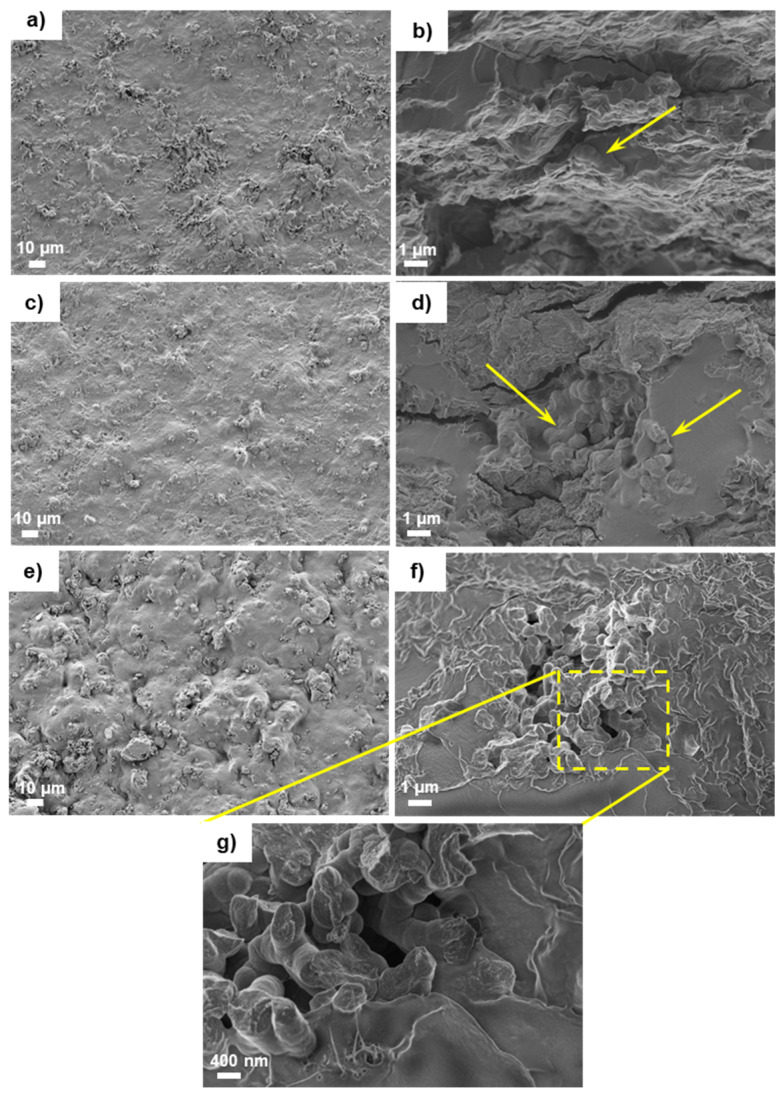
HR-FESEM photomicrographs of surface and impact fracture of semi-IPN PHBV/PVA with different percentage of PPy nanoparticles: (**a**) Surface of PHBV/PVA E2 composite; (**b**) Cross-section of PHBV/PVA E2 composite; (**c**) Surface of PHBV/PVA E10 composite; (**d**) Cross-section of PHBV/PVA E10 composite; (**e**) Surface of PHBV/PVA E15 composite; (**f**) Cross-section of PHBV/PVA E15 composite; (**g**) PPy nanoparticles embedded within the PHBV/PVA E15 composite in the cross-section at higher magnification. Arrows in (**b**) and (**d**) indicate PPy nanoparticles embedded in the matrix.

The images from the surface and cross-section of the PVBV/PVA semi-IPN samples with different percentages of PPy nanoparticles are included in Figure 2. The photomicrographs show that the surface morphology changes slightly after the addition of PPy nanoparticles. As the nanoparticle load content rises, the surface roughness increases progressively (Figure 2a,c,e, with 2%, 10% and 15% of PPy nanoparticles respectively), although the nanocomposite surface appears fully covered by the polymer matrix. In Figure 2b,d, showing the cross-section of the semi-IPN after adding 2% and 10% of PPy nanoparticles, the nanoparticles can be seen tightly embedded in the polymer matrix (see the arrows), forming a uniform structure. However, in the composite with the highest PPy nanoparticle content (15%) the particles tended to form aggregates with some voids (Figure 2f,g), suggesting the need for a stronger PPy dispersion mixing at high PPy nanoparticle contents.

**Figure 3:**
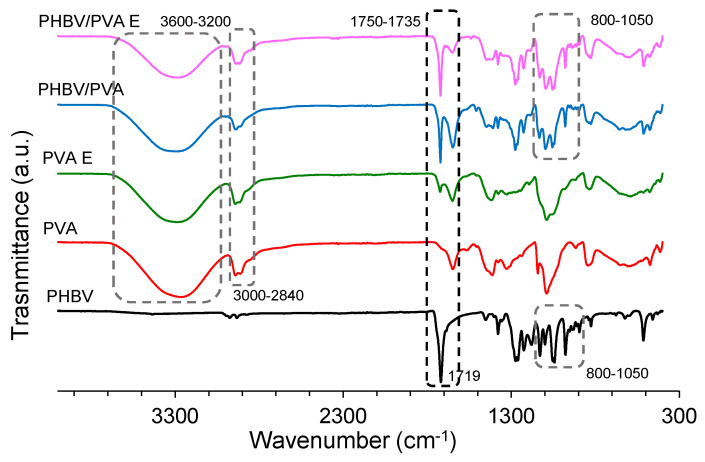
Fourier transform infrared spectroscopy (FTIR) spectra in the region of 4000–300 cm^−1^. PHBV, PVA, PHBV/PVA, PVA E and semi-IPN PHBV/PVA E.

FTIR spectra of PHBV/PVA and the semi-IPN PHBV/PVA are shown for comparison in Figure 3 together with neat PVA, PVA crosslinked with GA and PHBV as reference.

For neat PVA, the spectrum reveals the major vibrational peaks associated with poly (vinyl alcohol). The large bands between wavenumbers 3600 and 3200 cm^−1^ represent the stretching of OH groups due to the inter and intramolecular hydrogen bonds (H-bonds). The vibrational band between 2840 and 3000 cm^−1^ is related to the stretching of the C–H bond in the alkyl group and the band between 1750–1735 cm^−1^ is related to C=O stretch. PVA crosslinking is produced by the formation of acetal bridges between the hydroxyl groups in PVA and the difunctional aldehyde molecule of GA [55]. After crosslinking PVA with GA, the reduction of the relative intensity of the O–H bands (3600–3200 cm^−1^) was verified, which is evidence that the reaction of the PVA with GA has occurred by forming acetal bonds [52,55,56]. The PHBV spectrum shows a band at 1719 cm^−1^, related to the C=O stretch of the ester group present in the molecular chain of highly ordered crystalline structure. The peaks between 800 and 1050 cm^−1^ are characteristic of the -C–O–C- stretching vibration [57,58].

The spectrum of the PHBV/PVA blend shows the characteristic peaks related to both PVA and PHBV, confirming their presence in the blend. A reduction of the intensity of the O–H bands between 3600–3200 cm^−1^ can be observed in the semi-IPN PHBV/PVA obtained after crosslinking the PVA chains, which is indicative of the reduced number of the hydroxyl groups after the crosslinking reaction and the formation of the acetal bonds.

**Figure 4:**
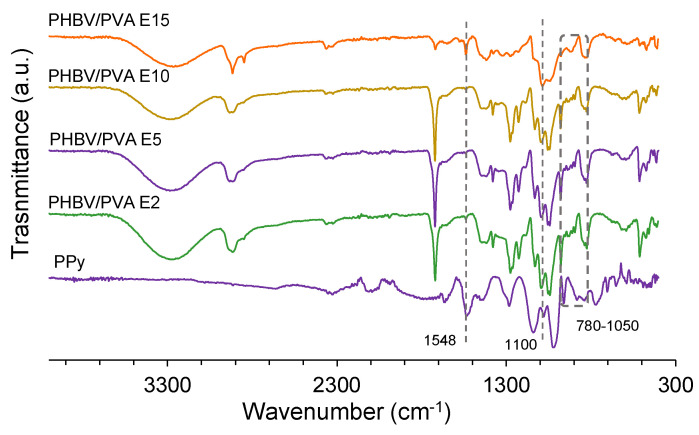
FTIR spectra in the region of 4000–300 cm^−1^. PHBV/PVA E with different percentages of PPy nanoparticles (2%, 5%, 10% and 15% referred to the mass sample).

The FTIR spectra of the composites after adding the PPy nanoparticles are depicted in Figure 4. The PPy particles show a peak at 1548 cm^−1^ due to the C=C stretching. The peak at 1100 cm^−1^ is related to C–C stretching and the peaks between 780 and 1050 cm^−1^ (dotted box) are associated with the torsion of the polypyrrole’s aromatic rings [59,60,61]. The composites revealed all the common peaks of the PHBV/PVA semi-IPN. The effect of the PPy nanoparticles in the PHBV/PVA network is more evident in composites with a concentration of 10% or higher, where the characteristic PPy peaks at 1100, 1548 cm^−1^ and the band between 780–1050 cm^−1^ can be identified. A slight reduction of the intensity of this band can also be observed as the percentage of nanoparticles increases. This behaviour suggests the presence of hydrogen bonding between the polymer matrix and the PPy nanoparticles.

### 3.2. Swelling Properties and Surface Wettability

**Figure 5:**
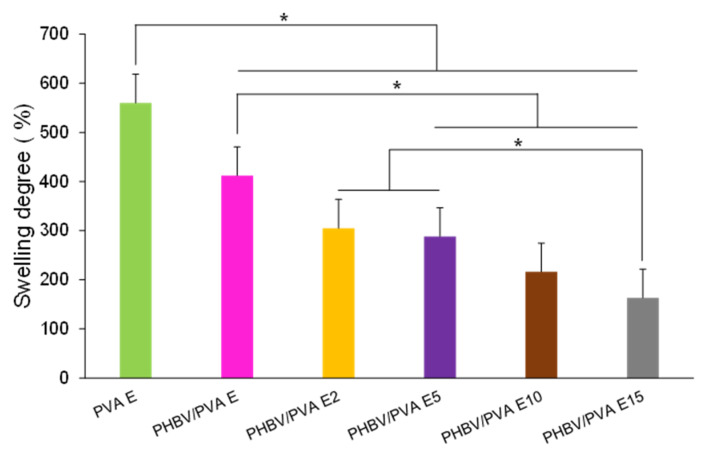
Swelling degree in equilibrium for PHBV/PVA E with different percentages of PPy nanoparticles ranging from 0 to 15 *wt/wt*%. PVA E is included as reference. Statistically significant differences (*p* < 0.05) are represented as (*).

The swelling degree of the crosslinked samples is depicted in Figure 5.

Crosslinked PVA shows the highest swelling degree (560% of its initial weight) due to the well-known hydrophilic nature of PVA [62]. Conversely, PHBV is a hydrophobic polymer, able to reduce the swelling ratio of pure hydrogels [63]. As expected, PHBV/PVA semi-IPN, with 30% of PHBV, shows a swelling degree ca. 400%, a value significantly lower than the crosslinked PVA. Adding PPy nanoparticles reduces the swelling degree in all the composites. Increasing the percentage of nanoparticles reduces the swelling degree monotonically, with significant differences with respect to the semi-IPN without nanoparticle at 5% or higher PPy concentrations. These results suggest that in addition to the PPy’s hydrophobic nature, PPy nanoparticles, embedded within the PHBV/PVA E matrix, entangle with PVA chains by hydrogen bonding (H-bonding) [64], forming a more compact structure with reduced swelling capacity [65]. This behaviour could be associated with the slight reduction of the band related to -OH bonds (3600–3200 cm^−1^) observed in the FTIR spectra when increasing the percentage of PPy nanoparticles (Figure 4). It is also worth noting that after the addition of the PPy nanoparticles, although the swelling capacity decreases, even in the semi-IPN with 15% nanoparticles, the swelling capacity is above 150%, which indicates that the nanocomposites retain the water absorption capacity, preserving their hydrogel properties.

The surface wettability, obtained from static water contact angle measurements, is included in Table 2. Contact angle of PHBV (~103°) and PVA with (~71°) are included as reference. The 30/70 PHBV PVA blend shows an increase in the contact angle, consistent with the presence of the hydrophobic chains of PHBV. As expected, crosslinking PVA increase the contact angle, both for neat PVA and the blend PHBV/PVA.

The incorporation of the PPy nanoparticles modifies the surface wettability. The contact angle increases with the percentage of nanoparticles, which have a hydrophobic nature. Compared to the semi-IPN without nanoparticles, the nanocomposites have a rougher surface (Figure 2) which may also slightly increase the WCA. These results are in good agreement with the swelling assay; the swelling degree decreases in the nanocomposites with incorporated PPy nanoparticles, which in turn present higher values of WCA.

### 3.3. Thermal Properties

Differential scanning calorimetry and thermogravimetry were used to get further insight into the thermal behaviour and degradation of the PHBV/PVA/PPy system.

#### 3.3.1. Differential Scanning Calorimetry (DSC).

##### Thermal Behaviour of PHBV/PVA Blends and Semi-IPN PHBV/PVA

**Figure 6:**
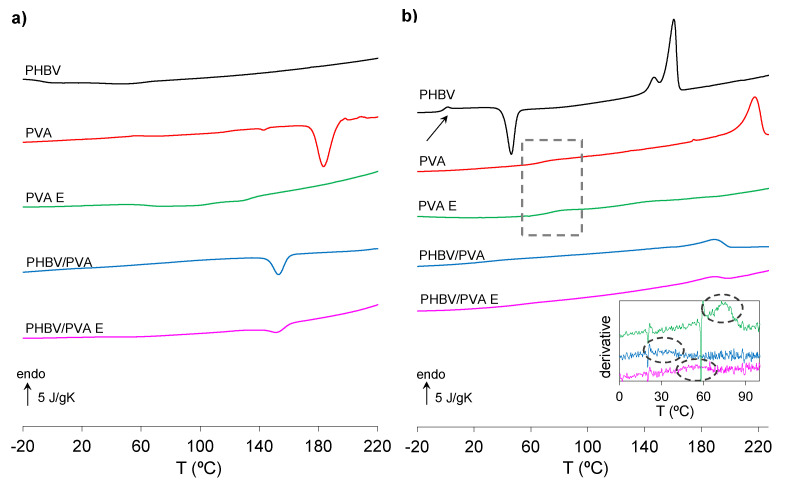
Differential scanning calorimetry (DSC) thermograms at a rate of 20 °C/min of neat PHBV and PVA, PHBV/PVA blend and PHBV/PVA E. (**a**) Normalized heat flow (*c_p_)* on cooling. (**b**) Normalized heat flow (*c_p_)* on heating. Arrows and rectangle mark the glass transition process. The inset shows the temperature derivative of the heat capacity from 0 to 100 °C (*dc_p_/dT)*. The arrow and dotted area in (**b**) and the inset indicate the glass transition process.

Among other different characterization techniques, DSC is one of the most widely used to determine miscibility of polymer blends and obtain further information on the molecular structure by studying the phase transformations. It is well established that two polymers are compatible with each other if they show a single glass transition temperature, *T_g_*, intermediate between the glass transition temperatures of both polymers. Figure 6 shows the DSC scan (cooling and heating) of PHBV/PVA, PVA E and PHBV/PVA E samples in which PVA was crosslinked forming a network and a semi- IPN network respectively, together with the control polymers (neat PHBV and PVA).

The glass transition and melting and crystallization processes can be observed in the thermograms. The 30/70 PHBV/PVA blend shows only one single *T_g_* (see the inset of Figure 6, where the inflexion point related to the glass transition is transformed into a maximum in the derivative*)* positioned in between the *T_g_* of the neat polymers (Table 3) showing the blends’ good compatibility and miscibility. The experimental values of *T_g_*, Δ*c_pi_* and Δ*T_g_*, obtained from the heating scan (Figure 6b), are enlisted in Table 3.

The Couchman and Karasz (CK) equation is a thermodynamic approach for predicting the glass transition temperature of blends [66]:

(3) $$T_{g}=\;\frac{\omega_{1}\Delta c_{p1}T_{g1}+\omega_{2}\Delta c_{p2}T_{g2}}{\omega_{1}\Delta c_{p1}+\omega_{2}\Delta c_{p2}}$$

where *T_g_* is the glass transition of the blend, *ω_i_* is the weight fraction of the component *i* and Δ*c_pi_* is the specific heat capacity increment at the glass transition.

The *T_g_* obtained from the PHBV/PVA blend according to CK prediction is 48.5 °C, which differs from that obtained experimentally (32.8 °C), suggesting interactions between the components in the blend not considered in Equation (3). Therefore, it is more appropriate to consider the Kwei equation [67]:

(4) $$T_{g}=\;\frac{\omega_{1}T_{g1}+k\omega_{2}T_{g2}}{\omega_{1}+k\omega_{2}}+q\omega_{1}\omega_{2}$$

where the parameter *k* can be obtained as the quotient between Δ*c_p_*_2_ and Δ*c_p_*_1_ and is related to the unequal contribution of each component in the mixture to the final *T_g_*. The term *qω*_1_*ω*_2_ represents the deviation of the glass transition temperature of the mixture from the linear weighted average of *T_g_*_1_ and *T_g_*_2_ and account for strong specific interactions within the mixture. The parameter *q* is used to model the effect of interaction between the components, such as H-bonding [68].

The value of *k* = 1.1, obtained from the Kwei equation (Equation (4)) for the PHBV/PVA blend, suggests that PVA is dominant in its contribution to the *T_g_* in the mixture. The Kwei equation provides a good fit of the experimental value of *T_g_* with *q* = −81.905. As indicated previously, *q* is related to the strength of specific interactions, reflecting the balance between the breakdown of self-association and formation of inter-associations between the two components. The negative *q* obtained indicates that the breaking of self-association (such as H-bonding between PVA molecules) reduces the *T_g_* more than the formation of H-bonds between PHBV and PVA can increase it [69].

PVA was chemically crosslinked to achieve aqueous stability and avoid the dissolution of the hydrophilic component in the blend, leading to a semi-IPN structure. As expected, *T_g_* increases in the crosslinked PVA sample (PVA E), from 69.2 to 74 °C, because of the reduced mobility of the chains imposed by the crosslinking. In the PHBV/PVA semi-IPN the glass transition process is difficult to detect, although it can be identified (as a maximum) in the derivative curves (*dc_p_/dT* vs. *T)* depicted in the inset of Figure 6b in the heating scan. The *T_g_* increases considerably with respect to the PHVB/PVA blend (from 32.8 to 49 °C), in a similar way as the crosslinked PVA (PVA E sample), whose *T_g_* increases from 69.2 to 74 °C. In the semi-IPN, both, the restricted of mobility in the PVA chains imposed by the crosslinking and the more rigid environment that surrounds the PHBV chains produced by the PVA network lead to a significant increase in the *T_g_*. (Table 3).

Both PHBV and PVA are semicrystalline polymers. The thermograms depicted in Figure 6a show that neat PHBV exhibits no crystallization in the cooling ramp, but an exotherm crystallization (cold crystallization process) can be observed during heating (46.7 °C), followed by melting (189.6 °C) (Figure 6b). Neat PVA shows a crystallization process and subsequent melting in the cooling and heating ramp at 183.5 °C and 217.5 °C, respectively. The degree of crystallinity of neat polymers was estimated by using Equation (5).

(5) $$X_{c}=\;\frac{\Delta H_{f}}{\Delta H_{f}^{0}}$$

where $\Delta H_{f}$ is the enthalpy of fusion of the samples and $\Delta H_{f}^{0}$ the enthalpy of fusion of the totally crystalline PHBV and PVA. Considering $\Delta H_{PVA}^{0}$ = 158 J/g [70] and $\Delta H_{PHB}^{0}$ = 132 J/g (assuming that only poly-hydroxybutyrate (PHB) crystals are produced in the PHBV copolymer due to the low percentage of hydroxyvalerate copolymer (2%)) [71], the degree of crystallinity of PHBV and PVA are 0.56 and 0.44 respectively (Table 3).

Crosslinked PVA (PVA E) shows no crystallization or melting process, indicating that crystallization was hampered by the crosslinking. The thermograms of the PHBV/PVA blend (Figure 6) show the presence of a crystallization process during the cooling followed by the subsequent melting on heating. In binary miscible semicrystalline polymer mixtures, the phase structure and morphology are affected by the crystallization processes of the components, determined by their different melting temperatures and ability to crystallize [72]. The PHBV/PVA blend under study shows a single crystallization peak (cooling) and melting peak (heating), and no cold crystallization was observed. The crystallization (153.9 °C) and melting (189 °C) temperatures are between those of the neat polymers, so the one with higher melt temperature, PVA, crystallizes, whereas the low melt-temperature component, PHBV, acts as a temporary amorphous diluent [72]. As only PVA crystallizes, the degree of crystallinity of the PHBV/PVA can be estimated (Equation (6)) from the enthalpy of fusion (∆*H_f_*), the PVA weight fraction in the sample (ω_2_), and the enthalpy of fusion of the total crystalline PVA.

(6) Xc= ΔHfω2ΔHPVA0

The degree of crystallinity in the blend, *X_c_*, is 0.16 (Table 3), lower than neat PVA, indicating that PHBV hampers PVA crystallization

Since the crosslinked PVA (sample PVA E) does not crystallize, it would initially seem that in the semi-IPN PHBV/PVA, any process related to crystallization or melting should be related to the PHBV component. But there are important differences between the thermograms of the semi-IPN and neat PHBV, which shows an exotherm crystallization process and subsequent melting during the heating, both at much lower temperatures than PVA crystallization and melting. In addition, the scan from the semi-IPN PHBV/PVA E and the blend PHBV/PVA are very similar, although the amplitude of both crystallization (endotherm) and melting peaks are smaller. Therefore, these results suggest that the processes observed are the crystallization and subsequent melting of the PVA component. Crystallization and melting processes of the semi-IPN are weaker than in the blend due to the crosslinking of PVA, but still appear. The PVA chains, with reduced mobility imposed by the crosslinking, still are able to crystallize (*X_c_* = 0.09), although to a much lesser extent than in the blend.

##### Thermal Behaviour of Semi-IPN PHBV/PVA with PPy Nanoparticles

**Figure 7:**
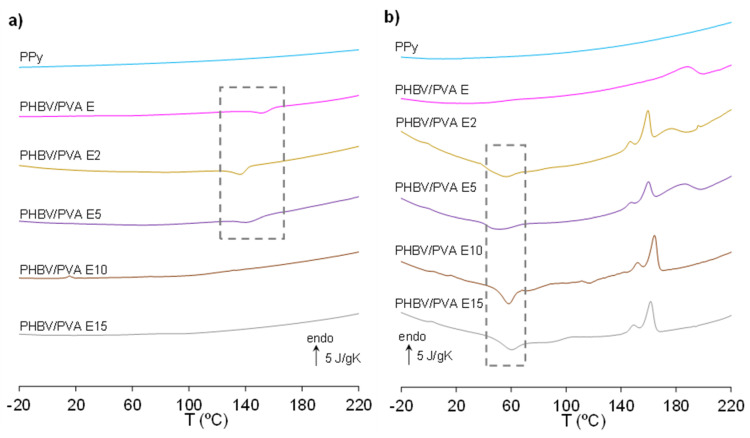
DSC thermograms at a rate of 20 °C/min of PHBV/PVA E with different percentages of PPy nanoparticles ranging from 0 to 15 *wt/wt*%. (**a**) Normalized heat flow on cooling and (**b**) normalized heat flow on heating. Rectangles mark endo and exotherm crystallization peaks.

DSC thermograms during cooling and heating after the inclusion of PPy nanoparticles are shown in Figure 7.

The PPy nanoparticles are also included in the figure, in which no *T_g_* was found in the temperature range analysed. The thermograms of PHBV/PVA semi-IPN composites with 2%, 5%, 10% and 15% PPy nanoparticles do not provide any thermal events that can be related to *T_g_*, although crystallization (both endotherm and exotherm) and melting processes could be observed, indicating that the presence of the nanoparticles influences the overall crystallization of the semi-IPN matrix. The temperatures related to crystallization and melting are listed in Table 4. Samples with 2% and 5% of PPy nanoparticles show both a crystallization process on cooling, close to the crystallization peak in the semi-IPN without PPy nanoparticles (doted area in Figure 7a) and a cold crystallization process during the heating in the same temperature range as PHBV (doted area in Figure 7b). The melting process, with several peaks, can be observed in a wide area from 140 to 200 °C. These results suggest that after the incorporation of small percentages of nanoparticles, PVA crystallization remains and the PPy nanoparticles act as nucleating agents for PHBV. The wide melting process suggest the melting of PHBV at lower temperature (the first two peaks) followed by the melt of PVA. The presence of these two peaks related to PHBV melting seems to indicate that PPy nanoparticles induce heterogeneous nucleation and lead to the formation of different sized crystals that melt at different temperatures, as has been reported for other nanoparticle-based fillers [73,74]. However, in samples with 10% and 15% of PPy nanoparticles, the crystallization peak related to PVA does not appear in the cooling scan and the cold crystallization peak related to PHBV is higher, indicating that when the amount of PPy nanoparticles increases, the PVA crystallization is prevented (maybe due to interactions between PVA chains and PPy nanoparticles), while the PHBV chains are able to crystallize to a larger extent due to the PPy nanoparticles acting as nucleating agents and the absence of PVA crystals. The degree of crystallinity (Table 3 and Table 4) related to PVA (X*c-PVA)* does not change significantly after the incorporation of 2% and 5% of PPy nanoparticles. However, the degree of crystallization related to PHBV (*Xc-PHBV)* increases from 0.31 to 0.42, as the percentage of PPy nanoparticles rises.

#### 3.3.2. Thermal Degradation Properties

**Figure 8:**
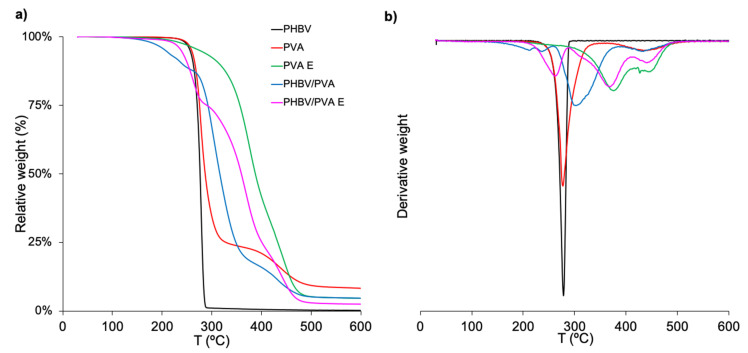
Thermogravimetry results of neat PHBV and PVA, PVA E, 30/70 PHBV/PVA blend and semi-IPN PHBV/PVA E. (**a**) Relative weight loss and (**b**) first derivative of the weight loss as a function of temperature (DTG).

**Figure 9:**
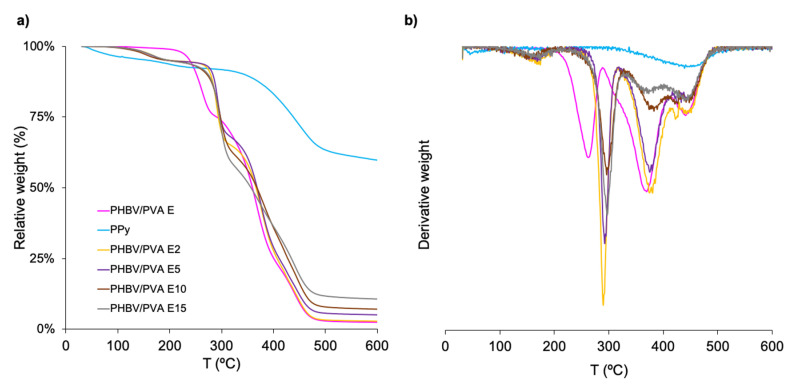
Thermogravimetry results of neat PPy nanoparticles and semi-IPN PHBV/PVA E with different percentages of PPy nanoparticles. PHBV/PVA E is included as reference. (**a**) Relative weight and (**b**) first derivative of the weight loss as a function of temperature (DTG).

The samples’ thermal degradation and thermal stability were determined by TGA analysis. The relative weight loss and the derivative of the weight loss as a function of temperature (DTG) curves in the range from 30 to 600 °C (see Figure 8 and Figure 9). The decomposition temperatures at which the weight loss is 50% (*T_d-50%_*) are indicated in Table 3 and Table 4.

##### PVA/PHBA Blend and PHBV/PVA Semi-IPN

Neat PHBV shows a unique degradation stage, starting at 251 °C (*T_onset_*) (Figure 8a). The 50% weight loss decomposition temperature is 276.1 °C, and at 290 °C the polymer is almost completely degraded, in good agreement with previous results [75,76]. It has been reported that PHBV is thermally unstable at temperatures above 250 °C, with hydrolysis and chain scission, which leads to the formation of crotonic acid [76]. PVA decomposition has been reported to be in three stages, with the one produced at temperatures between 50–130 °C related to moisture vaporization, followed by two additional degradation processes at higher temperature [77,78]. Figure 8a shows two-stage degradation depicted as two peaks in the DTG curve (Figure 8b), with no evidence of moisture (the samples were previously vacuum-dried, so there was no moisture vaporization is not observed). The first degradation process, at temperatures higher than 200 °C, is mainly due to the dehydration of hydroxyl groups and the formation of some volatile compounds followed by hydrocarbon products degradation, while the last stage, above 400 °C, involves the breakage of the main chain. The 50% loss decomposition temperature, reported in Table 3, is *T_d-50%_* = 286.8 °C.

The PHBV/PVA blend, also depicted in Figure 8, shows the initial degradation process, at temperatures lower than 250 °C, which may be related to the removal of moisture, mainly the water molecules bound to the PVA chains that remain trapped between the PHBV and PVA intertwined chains, even after previous vacuum drying. At higher temperature, the degradation profile contains the mixed characteristics of the blend components. However, the curve shifts to higher temperatures than those observed for both PHBV and PVA; *T_d-50%_* is ca. 316 °C, which can be attributed to hydrogen bonding interactions between the blend components that enhance thermal stability, as has been reported for other polymers interacting with PHBV [79,80,81].

The PVA E and the PHBV/PVA E (semi-IPN) crosslinked samples show that degradation happened at higher temperature (Figure 8a). PVA E has the highest shift with two degradation processes (*T_onset I_* = 220 °C and *T_onset II_* = 425 °C). The 50% weight loss temperature (*T_d-50%_* ∼ 387 °C) is 100 °C higher than neat PVA (Table 3), which denotes a significant increase in its thermal stability after crosslinking, as has been reported previously [82]. As expected, after crosslinking the PVA chains in the PHBV/PVA blend, the degradation profile of the semi-IPN shifted to higher temperatures, close to 44 °C (from 315.8 to 359.2 °C after crosslinking) in *T_d-50%_*, which indicates enhanced thermal stability of the PHBV/PVA semi-IPN.

##### Effect of Polypyrrole Nanoparticles in the Semi-IPN Thermal Degradation

The degradation behaviour in the PHBV/PVA semi-IPN before and after incorporating PPy nanoparticles is shown in Figure 9 together with the PPy thermal degradation profile, which has an initial weight loss (10%) below 220 °C, which can be related to moisture and dopant evaporation [54].

Degradation begins above 350 °C (Figure 9a), with a maximum around 450 ° (Figure 9b). At 600 °C, the final temperature of the scan that was performed, the weight loss is 40%, in good agreement with previous results [54,83,84]. Adding PPy nanoparticles to the PHBV/PVA semi-IPN does not significantly affect its degradation profile (Figure 9a). The first weight loss of around 10% can be related to the expulsion of moisture and the first stage of PPy degradation (dopant evaporation). The 50% weight loss temperature (Table 4) is in the same range as the semi-IPN matrix. At temperatures above 500 °C, the residual weight is in proportion to composition and rises with higher percentages of nanoparticles. These results indicate that the semi-IPN’s thermal stability is not significantly affected by the addition of PPy nanoparticles.

### 3.4. Electrical Behaviour

Nanoparticles of the conductive polymer PPy were initially introduced into PHBV/PVA semi-IPNs with the aim of increasing its conductive properties. The surface electrical conductivity is reported in Table 5 for neat PHBV and PVA and semi-IPN with and without PPy nanoparticles.

Semi-IPN conductivity without nanoparticles shows values in proportion to the composition. Adding PPy nanoparticles significantly increases conductivity, which is PPy concentration-dependent (from 2.79 mS/m for neat PHBV/PVA semi-IPN to 6.35 mS/m for 15% PPy composite), although the percolation threshold was not reached [83]. These values are in the same range as other electroactive biomaterials proposed for tissue engineering based on polymeric matrices that have shown an improved cellular response [85,86]. It can thus be concluded that PPy nanoparticles incorporation leads to a significant enhancement of the surface electrical conductivity of the nanocomposites.

## 4. Conclusions

Novel homogeneous PHBV/PVA semi-IPN hydrogels were prepared with different amounts of PPy nanoparticles by solvent casting using glutaraldehyde as PVA crosslinker. SEM images after PPy nanoparticle incorporation indicate that the particles were successfully embedded within the semi-IPN matrix. The FTIR and calorimetry results indicate good miscibility and compatibility between the components, with no phase separation. The parameters from the Kwei equation suggest the presence of strong specific interactions between the components, and that PVA is dominant in the glass transition process. The crystallinity of the semi-IPN PHBV/PVA, related to PVA, is less than that of the neat polymers and the PHBV/PVA blend, which can be associated with the mobility restrictions imposed by the PVA crosslinking. The addition of PPy nanoparticles increases the PHBV crystallization with the nanoparticles acting as a nucleation agent. The swelling assay indicates a significant reduction after adding PPy nanoparticles, even at low concentrations, indicating intermolecular interactions between the PPy nanoparticles and the semi-IPN hydrogel matrix. The hybrid hydrogel’s structural organization shows enhanced thermal stability, with no significant changes after adding PPy nanoparticles. The electrical conductivity is PPy concentration-dependent and increases significantly with the percentage of conductive polymer. Despite the results obtained, further studies are needed to demonstrate the potential applications of these stable electroactive hybrid hydrophilic networks in the biomedical field.

## 5. Patents

Patent Application No. CN201811517745, filed on 2 October 2020 by R.S.i.S., Á.S.-A., J.M.-M. and J.L.A.-C.

## Figures and Tables

**Table 1:** Sample notation for the films based on poly(3-hydroxybutirate-co-3-hydroxyvalerate) (PHBV), poly (vinyl alcohol) (PVA) and polypirrole (PPy).

Identification	Sample Description
PHBV	100% PHBV
PVA	100% PVA
PVA E	100% crosslinked PVA
PHBV/PVA	30% PHBV/70% PVA blends
PHBV/PVA E	30% PHBV/70% PVA semi-IPN
PPy	PPy nanoparticles
PHBV/PVA E2	30% PHBV/70% PVA semi-IPN with 2% PPy
PHBV/PVA E5	30% PHBV/70% PVA semi-IPN with 5% PPy
PHBV/PVA E10	30% PHBV/70% PVA semi-IPN with 10% PPy
PHBV/PVA E15	30% PHBV/70% PVA semi-IPN with 15% PPy

**Table 2:** Water contact angle measurement of PHBV/PVA blend, semi-IPN and composites with different percentages of PPy nanoparticles. PHBV and PVA (with and without crosslinking) are included as reference.

Sample	Contact Angle (°)
PHBV	103.2 ± 8.7 (◊)
PVA	70.8 ± 5.1 (*)
PVA E	83.3 ± 2.0 (*)
PHBV/PVA	74.7 ± 3.1 (**)(◊)
PHBV/PVA E	93.5 ± 6.3 (**)(♦)(◊)
PHBV/PVA E2	96.3 ± 2.7
PHBV/PVA E5	100.1 ± 5.9
PHBV/PVA E10	103.3. ± 2.7 (♦)
PHBV/PVA E15	109.7 ± 2.7 (♦)

(*)(**)(♦)(◊) Significant differences (*p* < 0.05) between samples.

**Table 3:** Glass transition temperature (*T_g_*), width of the glass transition *(*∆*T_g_*), heat capacity increment at the glass transition (∆*C_p_*), crystallization (*T_c_*) and melting (*T_m_*) temperature, enthalpy of fusion (Δ*H_f_*), degree of crystallinity PHBV (*X_c_ PHBV*) and PVA (*X_c_ PVA*) and 50% weight loss decomposition temperature (*T_d-50%_*) for neat PHBV and PVA, crosslinked PVA, PHBV/PVA blend and semi-IPN PHBV/PVA.

Sample	*T_g_* (°C)	Δ*T_g_* (°C)	Δ*C_p_* (J/g °C)	*T_c_* (°C)	*T_m_* (°C)	Δ*H_f_* (J/g)	*X_c_* PHBV	*X_c_* PVA	*T_d-50%_* (°C)
PHBV	0.77	3.67	0.5	46.7(*)	160.5	73.5	0.56	-	276.1
PVA	69.2	13.2	0.55	183.5	217.5	64.4	-	0.44	286.8
PVA E	74	36	2.05	-	-	-	-	-	386.9
PHBV/PVA	32.8	33.3	0.54	154	192.4	17.32	-	0.16	315.8
PHBV/PVA E	49.2(**)	-	-	151.5	188.2	11.2	-	0.09	359.2

(*) Cold crystallization. (**) Obtained from the derivative curve (*dc_p_/dT* vs. *T*).

**Table 4:** Crystallization (*T_c_*) and melting (*T_m_*) temperature, enthalpy of fusion (Δ*H_f_*), degree of crystallinity PHBV (*X_c_ PHBV*) and PVA (*X_c_ PVA*) and 50% weight loss decomposition temperature (*T_d_-_50%_*) for semi-IPN PHBV/PVA with different percentages of PPy nanoparticles.

Sample	*T_c_* (°C)	*T_m_* (°C)	Δ*H_f_* (J/g)	*X_c_* *PHBV*	*X_c_* *PVA*	*T_d-50%_* (°C)
PHBV/PVA E2	137.5/53.5(*)	140–200	21.9	0.31	0.09	366.3
PHBV/PVA E5	141.7/51.2(*)	140–200	22.9	0.36	0.08	368.3
PHBV/PVA E10	58.4(*)	140–170	16.4	0.45	-	368.5
PHBV/PVA E15	59.9(*)	140–170	14.0	0.42	-	356.8

(*) Cold crystallization exotherm (heating scan).

**Table 5:** Surface electrical conductivity of neat PHVA, PVA, PHBV/PVA 30/70 network and composites with from 2 up to 15% of PPy nanoparticles.

Sample	*σ_s_* (mS·m^−1^)
PHBV	2.38 ± 0.05
PVA	3.55 ± 0.14
PHBV/PVA E	2.79 ± 0.03
PHBV/PVA E2	3.48 ± 0.06(*)
PHBV/PVA E5	3.76 ± 0.04(*)
PHBV/PVA E10	4.93 ± 0.12(*)
PHBV/PVA E15	6.35 ± 0.15(*)

(*) significant difference (*p* < 0.05) with PHBV/PVA E.

## Data Availability

Please refer to suggested Data Availability Statements in section “MDPI Research Data Policies” at https://www.mdpi.com/ethics.
